# Acceptability and potential impact on uptake of using different risk stratification approaches to determine eligibility for screening: A population‐based survey

**DOI:** 10.1111/hex.13175

**Published:** 2020-12-02

**Authors:** Juliet A. Usher‐Smith, Laragh L. W. Harvey‐Kelly, Sabrina H. Rossi, Hannah Harrison, Simon J. Griffin, Grant D. Stewart

**Affiliations:** ^1^ The Primary Care Unit Department of Public Health and Primary Care University of Cambridge Cambridge UK; ^2^ University of Cambridge School of Clinical Medicine, Addenbrooke’s Hospital Cambridge UK; ^3^ Department of Oncology University of Cambridge, Addenbrooke’s Hospital, Cambridge Biomedical Campus Cambridge UK; ^4^ Department of Surgery University of Cambridge, Addenbrooke’s Hospital, Cambridge Biomedical Campus Cambridge UK

**Keywords:** acceptability, public attitudes, risk stratification, screening

## Abstract

**Background:**

Using risk stratification approaches to determine eligibility has the potential to improve efficiency of screening.

**Objectives:**

To compare the public acceptability and potential impact on uptake of using different approaches to determine eligibility for screening.

**Design:**

An online population‐based survey of 668 adults in the UK aged 45‐79 including a series of scenarios in the context of a potential kidney cancer screening programme in which eligibility was determined by age, sex, age and sex combined, a simple risk score (age, sex, body mass index, smoking status), a complex risk score additionally incorporating family history and lifestyle, or a genetic risk score.

**Outcome measures:**

We used multi‐level ordinal logistic regression to compare acceptability and potential uptake within individuals and multivariable ordinal logistic regression differences between individuals.

**Results:**

Using sex, age and sex, or the simple risk score were less acceptable than age (*P* < .0001). All approaches were less acceptable to women than men. Over 70% were comfortable waiting until they were older if the complex risk score or genetics indicated a low risk. If told they were high risk, 85% would be more likely to take up screening. Being told they were low risk had no overall influence on uptake.

**Conclusions:**

Varying the starting age of screening based on estimated risk from models incorporating phenotypic or genetic risk factors would be acceptable to most individuals and may increase uptake.

**Patient or Public Contribution:**

Two members of the public contributed to the development of the survey and have commented on this paper.

## 1 INTRODUCTION

Screening programmes seek to identify individuals with or at risk of developing disease to enable prevention or effective treatment. Most existing screening programmes are ‘one‐size‐fits‐all’ with eligibility determined by age and/or sex and screening tests and intervals standardized. However, within a population there is a wide range of risk of disease depending on individual factors, such as smoking status, body mass index (BMI), family history, lifestyle and genetics. There are, therefore, large differences in the absolute benefits of screening and potential harms that an individual is likely to experience.

Targeted or stratified screening, in which the age of first invitation, the choice of test and/or the screening interval are based on additional personal factors, has been proposed as a means of potentially improving efficiency.^1^, ^2^, ^3^, ^4^ However, moving from a system in which population screening is based only on age and/or sex, to one in which screening varies according to individuals’ risk of disease requires not only a valid and reliable means of estimating risk, but also consideration of many other aspects of implementation.^4^, ^5^, ^6^ In particular, any stratified screening programme must be acceptable to the public and uptake high. This is reflected in the English National Screening Committee's updated criteria published in 2015, in which there is increasing focus on the acceptability of screening programmes as a whole (as opposed to only screening tests) to both participants and society.^7^ Despite advances in the development of risk prediction models to estimate future risk, there remains little research on the views of the public towards introducing stratification into screening programmes.

Kidney cancer is the 9th most common cancer in men and the 14th commonest cancer in women worldwide, and the incidence is increasing.^8^ The disease is largely curable if identified at an early stage. However, over half of all patients with kidney cancer are asymptomatic at the early stages and over a quarter have evidence of metastases by the time of diagnosis when the five‐year survival rate is only 12%.^9^ This has led to international interest among the scientific and lay community in developing a potential screening programme for this ‘silent’ cancer.^10^, ^11^ As for other screening programmes, the cost‐effectiveness of any programme would be highly dependent on prevalence of kidney cancer in those screened.^12^ Targeted screening of higher‐risk individuals using established risk factors is, therefore, like to be the most cost‐effective strategy to maximize the benefits and reduce the harms of screening.^13^ The risk factors for kidney cancer overlap with those for many other cancers.^14^, ^15^, ^16^, ^17^ Within the context of a potential new screening programme for kidney cancer, we therefore compared the public acceptability and potential impact on uptake of using different individual level characteristics, either alone or within risk prediction models, to determine eligibility for screening in order to inform future risk stratification approaches within both new and existing screening programmes.

## 2 METHODS

### 2.1 Study design

An online population‐based survey.

### 2.2 Participants and recruitment

Participants were recruited through the Prolific platform (www.prolific.ac). Individuals aged 45‐79 years, with a Prolific approval rating (the proportion of prior studies completed by that participant that were judged by researchers as being of sufficient quality) over 95% were eligible to complete the survey. Inattentive participants were identified using a check question (‘*It is important that you pay attention in this study. Please tick “strongly disagree”*’) and excluded.^18^ We recruited a pragmatic sample of 1,021 adults. This analysis is based on data from 668 participants who correctly responded to the check question and resided in the UK.

### 2.3 Survey

This study reports the results from the section of the survey that focused on attitudes towards different approaches to defining eligibility for screening. Questions in this section were developed with input from patient and public representatives and informed by questions from a previous study exploring attitudes towards cessation of low‐value colorectal cancer screening that had been piloted using a think‐aloud approach.^19^ As in that study, we presented participants with a series of hypothetical screening scenarios. Before seeing the scenarios, participants were informed that a person's risk of developing kidney cancer depends on many factors and, specifically, that kidney cancer is more likely in older people, men, and people who smoke, are overweight or have a family history of kidney cancer. In each scenario that followed participants were then asked to imagine that they receive a letter inviting them to screening and justifying why they are being invited at that time. The first scenario used age alone to determine eligibility, with all individuals invited at age 60 because ‘*kidney cancer does not usually occur in younger people*’. The second used sex alone, with men being invited and women not because ‘*men are 2‐3 times more likely to develop kidney cancer than women*’ and the third used both age and sex, with men being invited earlier than women. In the fourth scenario, eligibility was determined using a risk calculator incorporating age, sex, BMI and smoking status, with participants at higher risk invited at younger ages. No indication was given on the threshold used to determine eligibility for screening. The fifth scenario added family history and lifestyle to the risk calculator, and the final, sixth, scenario was based on genetic risk. After each scenario, acceptability was assessed by asking participants to rate how reasonable they thought it was to use the information in that scenario to decide when individuals should be invited to screening and how comfortable they felt about that information being used. Responses were recorded on a 7‐point Likert scale from 1 ‘Not at all reasonable/comfortable’ to 7 ‘Extremely reasonable/comfortable’. After each scenario, participants were also asked how comfortable they felt with not being offered screening or having to wait until they are older if they were low risk, again on a 7‐point Likert scale. We also asked participants how acceptable it was to complete a questionnaire or provide a sample of blood or a cheek swab to enable estimation of their risk. Additionally, participants were asked how much being told they were at lower or higher risk would influence their decision to take up screening (on a 5‐point Likert scale from 1 ‘Much less likely to attend’ to 5 ‘Much more likely to attend’).

The survey also included questions on key personal characteristics: age, sex, ethnicity, BMI, education level, social class (classified as higher (ABC1) and lower (C2DE) based on the household's chief income earner's occupation category^20^, ^21^), personal history of cancer and family history of kidney cancer.

Full details of all the scenarios and questions are in Appendix S1.

### 2.4 Consent

Written online consent was obtained from each participant before they began the survey.

### 2.5 Analysis

Data were weighted by age and sex within the UK so that the sample profile matched those of people aged 45‐79 in the UK derived from the mid‐year population in 2018 obtained from the Office of National Statistics.^22^ All results presented are from weighted data.

To enable us to compare the acceptability of the six different approaches to determining eligibility for screening, we generated a single measure of acceptability on a scale from 1 to 7 by calculating the mean of how reasonable participants considered each approach and how comfortable participants were with that approach, with 1 indicating ‘not at all acceptable’ and 7 indicating ‘extremely acceptable’. To compare the overall acceptability of the different approaches at a population level, we first summarized the distribution of acceptability scores for each of the six scenarios. This was performed separately for men and women as the questions relating to the sex and age and sex scenarios were different between the two sexes. To enable us to compare the relative acceptability of the different approaches at an individual level while accounting for the multiple responses from each participant, we then performed a two‐level mixed‐effects ordinal logistic regression with the single measure of acceptability treated as an ordinal variable and responses nested within participant, adjusted for age, social class and level of education and stratified by sex. To further explore whether acceptability for each scenario was influenced by age, sex, social class or level of education, we additionally performed a series of multivariable ordinal logistic regression analyses with each approach separately as the dependent variable and age, sex, education level and social class as independent variables. We used the same approach to analyse how comfortable participants were with either not being screened or having to wait until they were older if they were low risk. In both cases, where data were missing for social class, participants with missing data were excluded from the regression models.

Responses from men and women were combined for analysis of the acceptability of data collection and the potential influence of being low or high risk on intention to take up screening. For both these outcomes, we summarized the data and then performed multivariable ordinal logistic regression with each outcome (acceptability and influence of being low or high risk on intention) as the dependent variable to explore differences with age, sex, education level and social class.

We report all regression analysis results as odds ratios (ORs) with 95% confidence intervals. All analyses were performed using Stata version 14^23^ with statistical significance set at *P* < .05.

## 3 RESULTS

The characteristics of the 668 participants are shown in Table 1. Participants were distributed evenly by age group (237 (35.4%) aged 45‐54, 203 (30.4%) 55‐64 and 228 (34.2%) 65 and over) and sex (343 (51.3%) were female). Almost all (98%) described their ethnicity as white, most (75%) were in social classes ABC1 and 285 (42.7%) had university level education. There were < 5% missing data for social class (n = 32/668, 4.8%), body mass index (n = 29/668, 4.3%) and family history of kidney cancer (n = 14/668, 2.1%). All other questions in the survey were completed by all participants.

**Table 1:** Characteristics of participants

Participant characteristic	Unweighted n (%) or mean (± SD)	Weighted n (%) or mean (± SD)
Age		
45‐54	383 (57.3)	237 (35.4)
55‐64	214 (32.0)	203 (30.4)
>65	71 (10.6)	228 (34.2)
Mean (± SD)	54.7 (± 7.0)	59 0.0 (± 8.4)
Sex (n, % female)	373 (55.8)	343 (51.3)
Social class		
ABC1	500 (74.9)	501 (75.0)
C2DE	136 (20.4)	131 (19.6)
Missing	32 (4.8)	37 (5.5)
University level education	288 (43.1)	285 (42.7)
Ethnicity (n, % White)	653 (97.8)	657 (98.4)
Self‐reported general health		
Excellent, very good, good	524 (78.4)	534 (79.9)
Fair, poor	144 (21.6)	134 (20.1)
Smoking status		
Non‐smoker	340 (50.9)	318 (47.6)
Ex‐smoker	236 (35.3)	257 (38.5)
Current smoker	92 (13.8)	93 (14.0)
BMI (kg/m^2^) (mean ± SD)	27.4 (± 5.8)	27.1 (± 5.3)
Missing	29 (4.3)	29 (4.3)
Previous diagnosis of cancer	34 (5.1)	44 (6.6)
Family history of kidney cancer		
Yes	19 (2.8)	17 (2.6)
No	635 (95.1)	636 (95.2)
Missing	14 (2.1)	15 (2.2)

### 3.1 Acceptability of using different approaches to determine eligibility for screening

**Figure 1:**
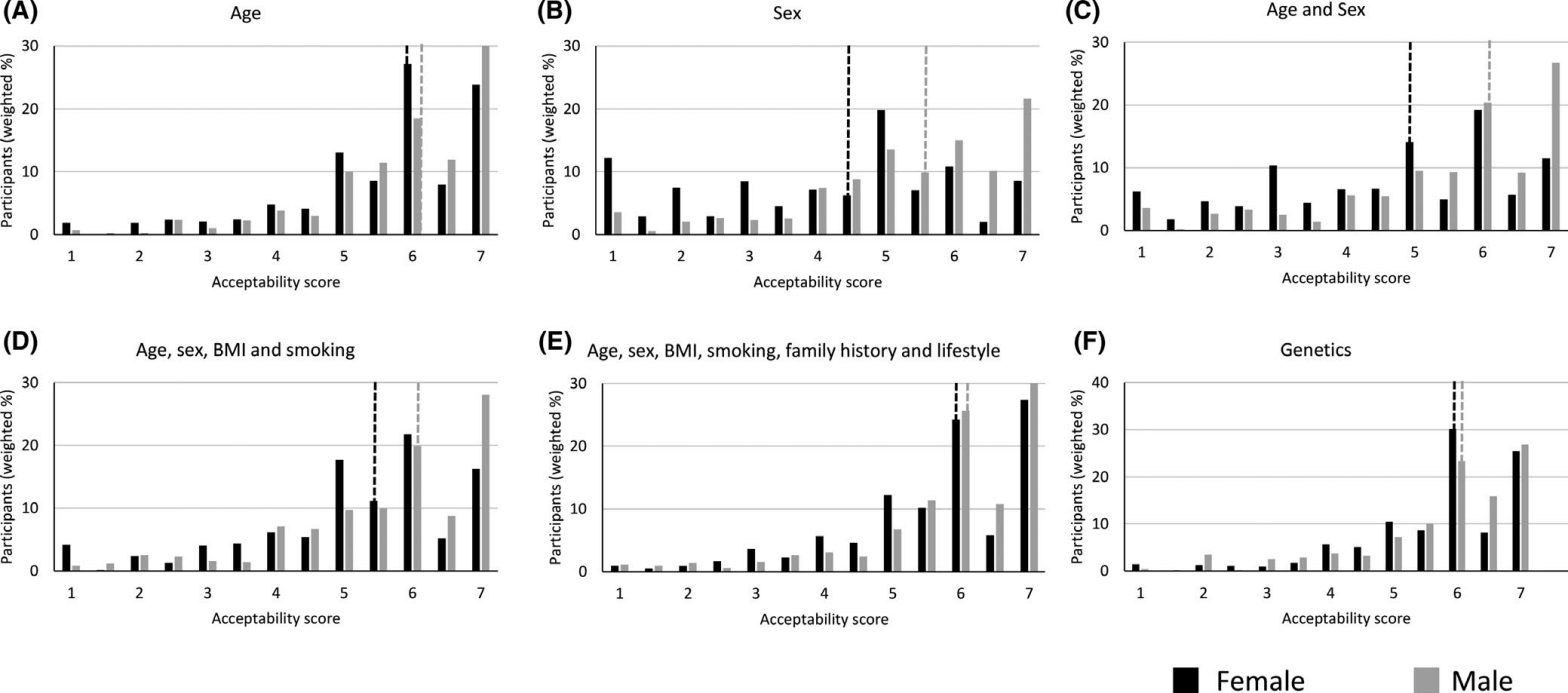
Acceptability of using either (A) Age, (B) Sex, (C) Age and sex, (D) Age, sex, BMI and smoking, (E) Age, sex, BMI, smoking, family history and lifestyle or (F) genetics to determine starting age of screening. Acceptability scores were measured on a Likert scale from 1 indicating not at all acceptable to 7 indicating extremely acceptable. Dotted lines indicate the median response

**Figure 2:**
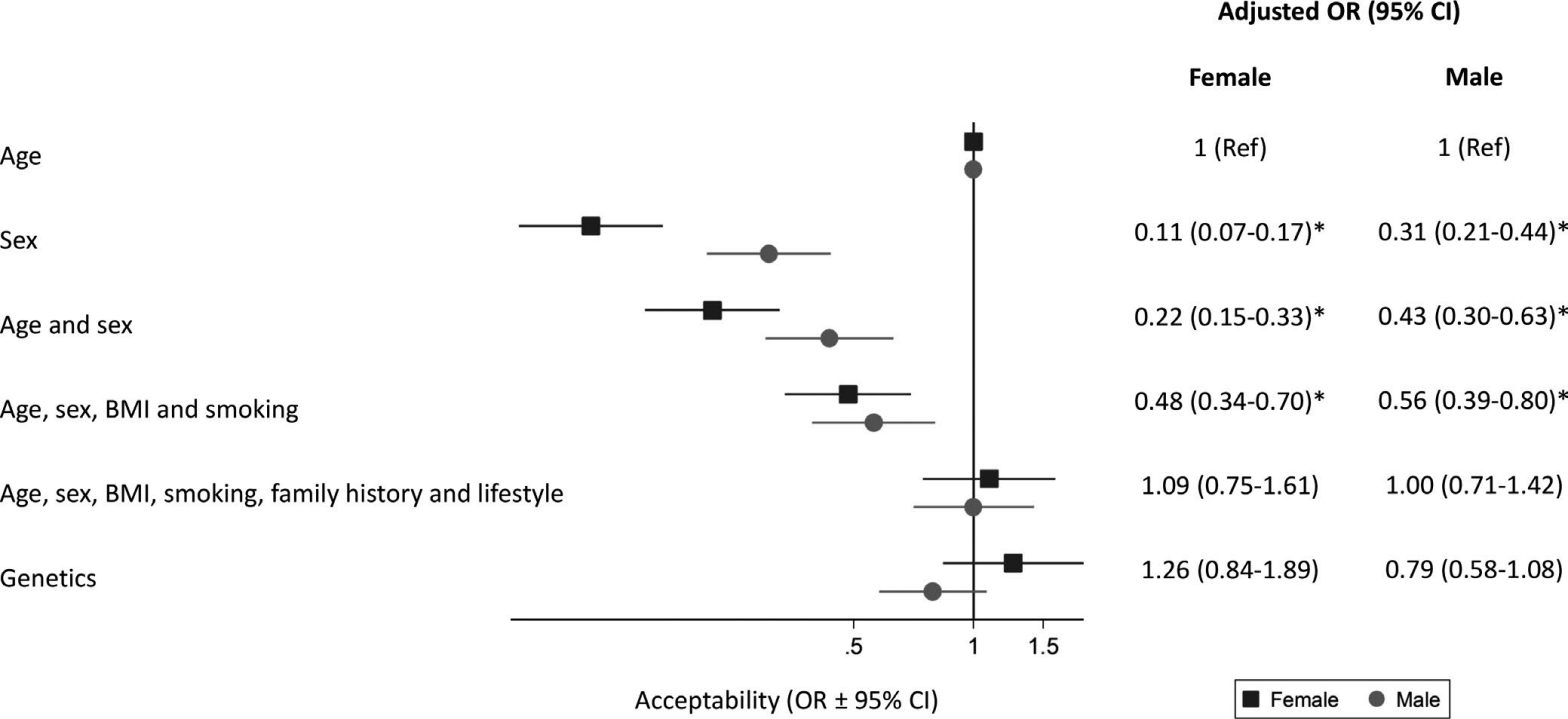
Odds (OR ± 95% confidence intervals) of considering using different approaches to determine the starting age of screening acceptable, adjusting for age, sex, level of education and social class and clustering of response by participant. The ORs represent the association between a 1‐point increase in acceptability (measured on a Likert scale from 1 indicating not at all acceptable to 7 indicating extremely acceptable) and the approach to determining eligibility. * *P* < .0001 compared with using age

Figure 1 shows the distribution of acceptability scores for men and women for different approaches to determine when individuals would become eligible for screening. Overall, 83% responded that it was acceptable (a score of ≥ 5 on the 7‐point Likert scale) to use age, the more comprehensive risk score or a genetic risk score. This percentage was lower for a simple risk score (74.2%), for age and sex (65.0%) and lowest for sex alone (58.9%). This pattern was also seen at individual participant level in the multi‐level ordinal regression analyses, with sex, age and sex, and the simple risk score significantly (*P* < .0001) less acceptable than age, the complex risk score or genetic risk. Acceptability was the same for age, the complex risk score and genetic risk for both male and female participants (Figure 2).

In multivariable analysis at population level (Table 2), using age, sex, age and sex, or the simple risk score were all more acceptable to men than women. Age, the more complex risk score and genetics were also more acceptable to participants over 65 than to younger age groups. Sex, and age and sex were more acceptable to participants with a university education than those with lower levels of education. No differences were seen with social class.

**Table 2:** Multivariable analysis of acceptability of using different approaches to determine eligibility for screening at population level. Odds ratios (OR) from multivariable ordinal logistic regression mutually adjusted for all factors in the table. Significant (*P* < .05) results are shown in bold

Factor	Age Multivariable OR (95% CI)	Sex Multivariable OR (95% CI)	Age and sex Multivariable OR (95% CI)	Simple risk score (age, sex, BMI and smoking) Multivariable OR (95% CI)	Complex risk score (age, sex, BMI, smoking, family history and lifestyle) Multivariable OR (95% CI)	Genetics Multivariable OR (95% CI)
Age						
45‐54	Ref (1)	Ref (1)	Ref (1)	Ref (1)	Ref (1)	Ref (1)
55‐64	1.25 (0.94‐1.67)	0.98 (0.75‐1.29)	1.06 (0.80‐1.41)	0.90 (0.67‐1.21)	1.23 (0.91‐1.68)	0.98 (0.72‐1.33)
>65	**2.40 (1.41‐4.08)**	1.24 (0.71‐2.17)	1.18 (0.70‐1.98)	1.66 (1.00‐2.74)	**2.22 (1.35‐3.64)**	**1.83 (1.13‐2.95)**
Sex						
Female	Ref (1)	Ref (1)	Ref (1)	Ref (1)	Ref (1)	Ref (1)
Male	**1.59 (1.11‐2.28)**	**3.15 (2.19‐4.54)**	**2.36 (1.64‐3.39)**	**1.78 (1.24‐2.55)**	1.43 (1.00‐2.04)	1.18 (0.83‐1.67)
Social class						
ABC1	Ref (1)	Ref (1)	Ref (1)	Ref (1)	Ref (1)	Ref (1)
C2DE	0.81 (0.49‐1.34)	1.02 (0.63‐1.65)	1.06 (0.64‐1.73)	0.85 (0.52‐1.37)	0.99 (0.63‐1.53)	1.07 (0.68‐1.67)
University education						
No	Ref (1)	Ref (1)	Ref (1)	Ref (1)	Ref (1)	Ref (1)
Yes	1.07 (0.74‐1.54)	**1.80 (1.21‐2.68)**	**2.04 (1.38‐3.02)**	1.16 (0.79‐1.71)	1.08 (0.74‐1.58)	1.08 (0.75‐1.55)

**Figure 3:**
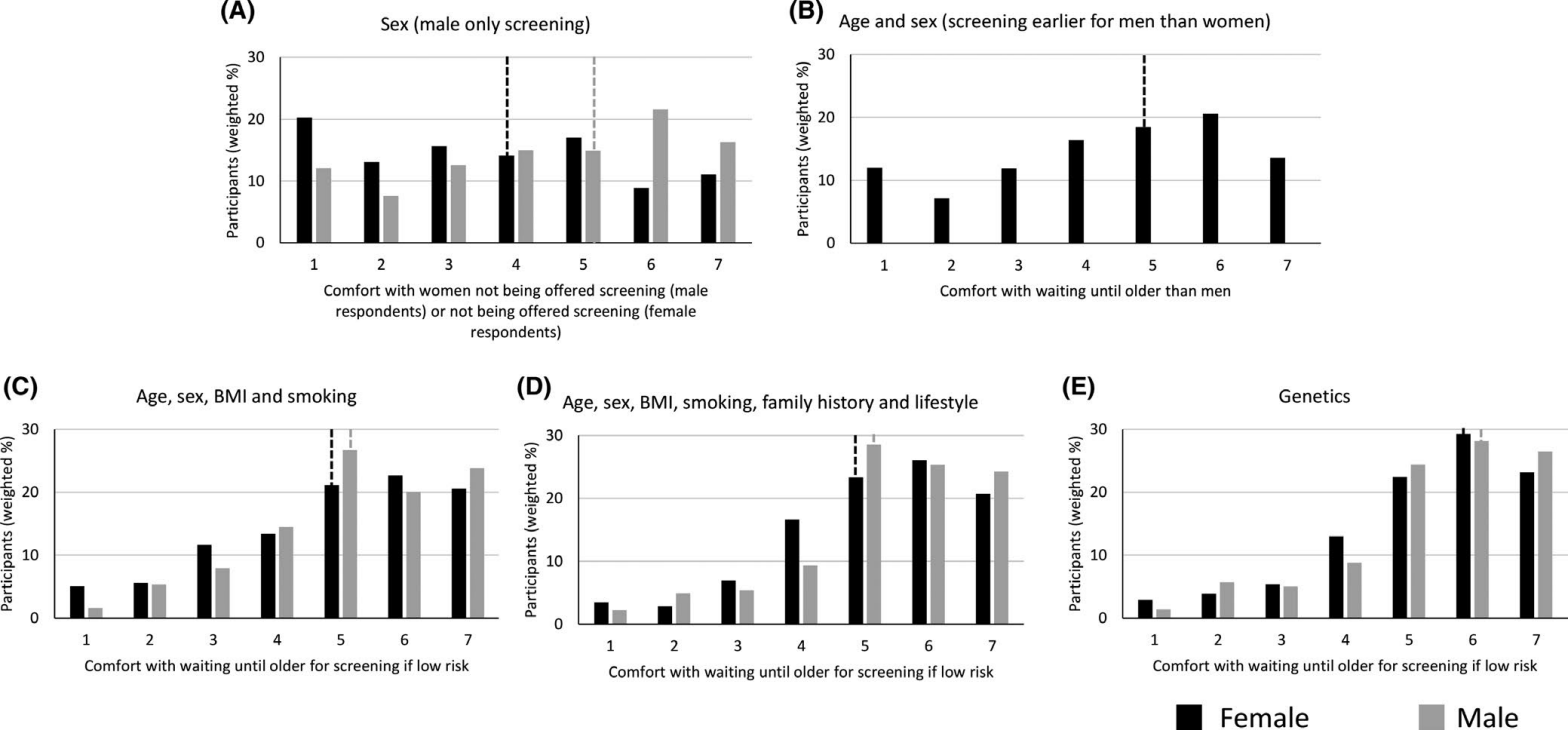
Comfort with waiting until older if low risk based on (A) Sex, (B) Age and sex, (C) Age, sex, BMI and smoking, (D) Age, sex, BMI, smoking, family history and lifestyle or (E) genetics to determine starting age of screening. Comfort scores were measured on a Likert scale from 1 indicating not at all comfortable to 7 indicating extremely comfortable. Dotted lines indicate the median response

**Figure 4:**
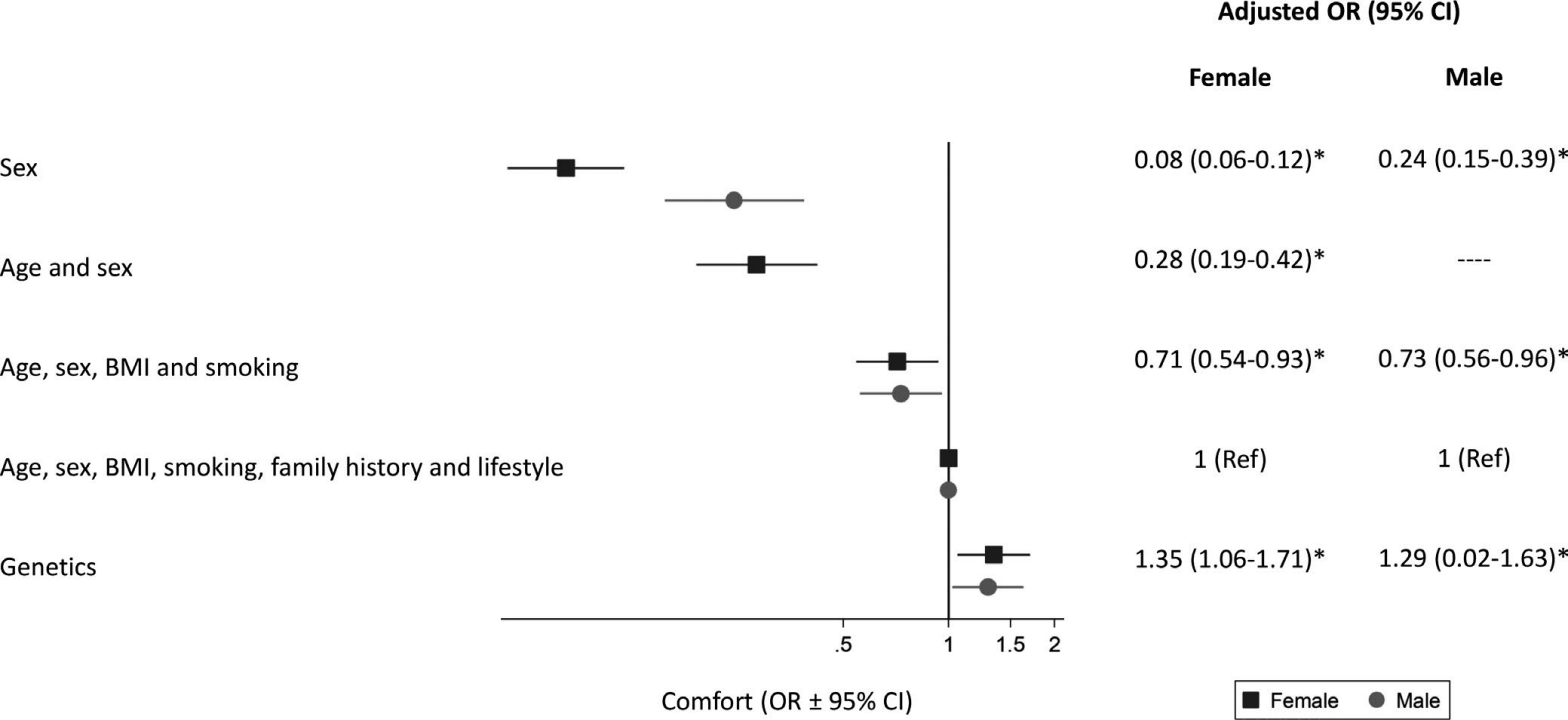
Odds (OR ± 95% confidence intervals) of being comfortable with waiting until older if low risk based on different characteristics, adjusting for age, sex, level of education and social class and clustering of response by participant. The ORs represent the association between a 1‐point increase in comfort (measured on a Likert scale from 1 indicating not at all comfortable to 7 indicating extremely comfortable) and the approach to determining risk. * *P* < .05 compared with using age, sex, BMI, smoking, family history and lifestyle

Similar patterns were seen when considering how comfortable participants would be either not being offered screening or having to wait until they are older if they were low risk (Figures 3 and 4). Both men and women were least comfortable with sex or age and sex alone being used to determine eligibility. In particular, only 36.9% of female participants were comfortable with not being offered screening at the same age as men. Men were more comfortable than women with the age one becomes eligible for screening being determined by sex (multivariable OR 2.05 (1.42‐2.96), Table S1) but 32.2% of men were still not comfortable (a score of 3 or less). Over 70% of both men and women were comfortable (a score of 5 or more) with waiting until they were older if they were low risk based on the complex risk score or genetics.

Participants over 65 were more comfortable than younger participants waiting until they were older if they were low risk based on the simple risk score (age, sex, BMI and smoking), the more complex risk score and genetics. Those with a university education were also more comfortable with men being screened and women not than those with lower levels of education. No differences were seen with social class (Table S2).

### 3.2 Acceptability of data collection to enable estimation of risk

40.3% (n = 269/668) and 46.9% (n = 313/668) responded that it was extremely acceptable to complete either a questionnaire or provide a sample of blood or cheek swab for genetic analysis, with 88% scoring 5 or higher on the 7‐point Likert scale and less than 6% scoring 3 or less (Figure S1). Participants aged over 65 years considered both methods of data collection more acceptable than those aged 45‐54 years (multivariable OR 2.34 (95% CI 1.39‐3.95) and 2.08 (1.20‐3.62) for questionnaire and genetic samples, respectively). No differences were seen with sex, university education or social class (Table S2).

### 3.3 Influence of being low or high risk on intention to take up screening

**Figure 5:**
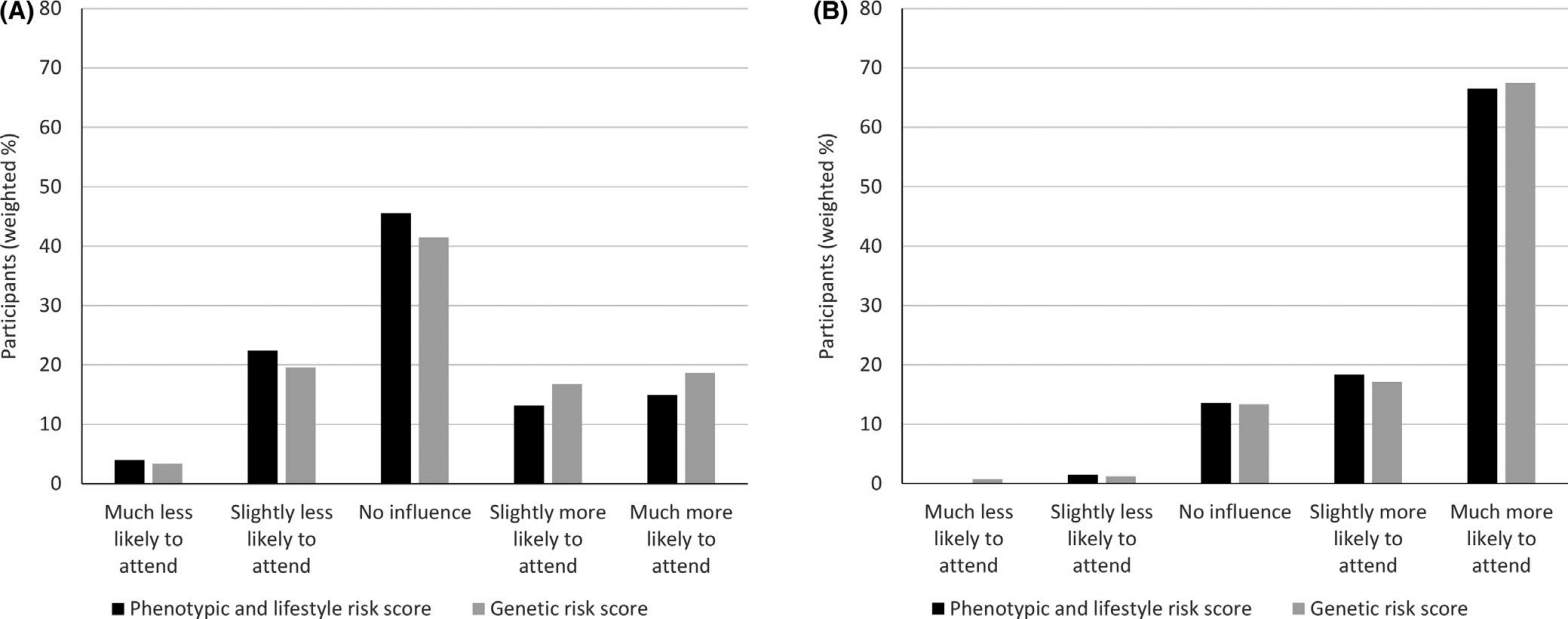
Influence of being (A) low risk and (B) high risk on likelihood of attending screening for both a phenotypic and lifestyle risk score and a genetic risk score

Almost half of participants responded that being told they are at low risk, based on a risk score incorporating either phenotypic and lifestyle factors or genetic risk factors, would have no influence on their intention to take up screening (45.6% for a phenotypic and lifestyle risk score and 41.5% for a genetic risk score) (Figure 5A). The remainder were approximately evenly split between those who would be less likely to attend and those who would be more likely to attend, with the mean intention 3.12 (SD 1.05) and 3.28 (SD 1.08) on a scale from 1 to 5. When asked about the influence of being told they are at high risk, 85% of participants reported they would be more likely to attend with both types of risk score and only 2% less likely to attend (Figure 5B). No differences were seen with age, sex, university education or social class.

## 4 DISCUSSION

Identifying strategies that are acceptable to the public has been recognized as one of the challenges to implementation of risk‐stratified screening.^2^, ^3^, ^4^, ^5^ In the context of a potential new screening programme for kidney cancer, we have shown that using risk models based on genetic risk factors or age, sex, BMI, smoking, family history and lifestyle to determine eligibility for screening is as acceptable as using age alone for both men and women. Furthermore, over 70% of participants were comfortable with waiting until they were older if they were low risk. In contrast, using sex or a combination of age and sex, in which only men (who are at higher risk of kidney cancer) are invited or men are invited at a younger age than women, was less acceptable than age alone, with up to half of women and a third of men not comfortable with using sex, and both approaches less acceptable to women than men.

### 4.1 Comparison with existing literature

To our knowledge, this is the first study to report the public acceptability of using different individual level characteristics either alone or within risk prediction models to determine initial eligibility for screening. A survey of veterans in the US explored attitudes towards using age or a risk score to decide when to stop colorectal cancer screening.^19^ They found that participants were divided over the use of both age or a risk score, with 22% and 24.3% respectively responding that using age or a risk score was not at all reasonable and 16.3% and 11.3% responding that each was extremely reasonable. Age was considered more reasonable overall than using a risk score in that study, although that may reflect a preference for status quo that has been reported in previous studies.^24^

Other studies have focused on attitudes towards changes in the frequency of screening. In general, these have found that members of the public are positive towards being offered more screening but are concerned about the possibility of less screening.^24^, ^25^, ^26^, ^27^ Although a significant proportion of participants in this study were uncomfortable about only men having screening and a significant proportion of women uncomfortable about having to wait until they were older than men for screening, most (>70%) were comfortable waiting until they were older to start screening if they were low risk when that risk was based on a risk score or genetic risk. It is possible that delaying the start of screening based on risk scores may be more acceptable than extending the interval between screening episodes.

The high levels of acceptability for either completing a questionnaire or providing a cheek swab or blood sample for genetic testing mirror the general support for cancer risk assessment seen in other screening contexts. In surveys of the general public, for example, 85% of women report that they would be likely to take up genetic testing for ovarian cancer risk,^28^ 94% of women would take up risk assessment for breast cancer^29^ and 94% of respondents were interested in knowing their risk of breast or prostate cancer.^30^ The greater acceptability among older participants seen in this study may reflect a more general greater familiarity and comfort with blood testing.

The finding that being told that they are low risk is unlikely to have a significant effect on subsequent uptake, while being told high risk might increase uptake, is also consistent with the findings from a UK‐based study in which women were given their risk of breast cancer in the context of breast cancer screening: re‐attendance was significantly higher for women told they were at high risk than usual re‐attendance rates but was not significantly lower for those told they were at low risk.^31^ This may reflect an overall enthusiasm for screening^32^ or just being invited may be sufficient for some individuals to consider they should take up screening, with information about their risk not being a factor in that decision. How much these changes in intention observed in this study might influence uptake is also not known. These findings, together with the wider body of literature concerning the impact of provision of personalized risk information,^33^, ^34^, ^35^ however, suggest that informing individuals of their risk is unlikely to substantially reduce overall screening uptake and may increase uptake.

### 4.2 Strengths and limitations

A key strength of this study is that the survey was informed by questions developed in a previous study^19^ using a think‐aloud approach and informed by input from patient and public representatives to maximize readability for participants. While we provided estimates of the relative risks of many of the risk factors included in the different scenarios, we did not, however, provide detailed quantitative estimates of the risks and benefits of screening in each scenario, accuracy of the risk models, or the population‐level impacts of inviting groups of individuals at different times. It is possible that the participants’ views may have been different had we presented this additional information. The wording also varied slightly between the individual scenarios. Specifically, in the age‐based scenario participants were informed that ‘*kidney cancer does not usually occur in younger people’* while in the scenarios including sex the participants were informed that ‘*kidney cancer is 2‐3 times more common in men than women’*. Additionally, in all the risk model–based scenarios no indication was given of the thresholds that might be used to categorize individuals as at higher or lower risk but participants were explicitly informed that the risk calculator is not 100% reliable. The more certain framing used in the age‐based scenario could explain in part the high levels of acceptability for using age alone to identify individuals for screening. The order of the questions may also have impacted on the results as the scenarios were not randomized and so information presented in earlier scenarios may have influenced how participants responded to later scenarios. Such context effects are thought to occur through a number of distinct cognitive processes, including priming effects, in which the beliefs around an earlier question trigger a similar response to later questions, and anchoring, in which information presented earlier influences comparative judgements about later questions.^36^ It is difficult to predict these effects^37^ and, in turn, how they may have impacted on our findings. Experimental studies suggest, however, that while question order effects may affect responses to individual items, they do not affect the relationships between responses or correlations with participant characteristics.^37^

Asking these questions in the context of a hypothetical new screening programme could also be considered a strength as it reduced the likelihood of our findings being biased by a preference for status quo screening. However, it is possible that the participants’ views may have been different if we had used scenarios within the context of screening programmes for other conditions such as colorectal, breast or prostate cancer. Although the risk factors included in this study are the same as the risk factors for other cancers^15^, ^16^, ^17^ and the strategies similar to those being considered for these existing screening programmes,^2^, ^38^, ^39^ our findings may not therefore be generalizable across all screening programmes.

Another strength is the large sample size that allowed us to explore differences in acceptability at both the population and individual level. However, to achieve this we used an online recruitment platform which potentially limits generalizability. In particular, members of the platform (Prolific) are experienced at completing online studies and their views may not be representative of the general population,^40^ particularly those with lower literacy levels and those without internet access. The demographics of members are also different from the general UK population. The main differences in our cohort compared with UK census data from 2011 were in social class and ethnicity: 74.9% of our participants reported being in the upper half of the social classes (ABC1), compared with 53.0% of the UK population^41^; and 97.8% reported being of white ethnicity, compared with 86% in the UK.^42^ The proportion with university education, number of current smokers and mean BMI were all similar to the UK population.^43^, ^44^, ^45^ To enable our population to reflect the age and sex distribution across the UK, we weighted the analysis by age and sex. While this application of weights makes the findings more generalizable to the UK population, it does not however, eliminate the risk of bias as the approach is equivalent to replacing members of under‐represented subgroups with replicates of participating members of the same subgroup. This approach also does not account for differences in social class or ethnicity, or for potential differences in attitudes between those registered with the online recruitment platform and the wider population.

## 5 CONCLUSION

This study suggests that changing the starting age of screening based on estimated risk from risk models incorporating either phenotypic or genetic risk factors would be acceptable to the majority of individuals and may lead to increased uptake. Further work is now needed to explore the wider social and ethical implications of risk stratified screening for society as a whole.

## CONFLICT OF INTEREST

GDS has received educational grants from Pfizer, AstraZeneca and Intuitive Surgical; consultancy fees from Pfizer, Merck, EUSA Pharma and CMR Surgical; Travel expenses from Pfizer and Speaker fees from Pfizer. All other authors have no financial disclosures or conflicts of interest with respect to the research, authorship, and/or publication of this article.

## AUTHORS’ CONTRIBUTIONS

All authors were involved in the design of the study. JUS completed data collection and initial analysis. All authors contributed to the final analysis and interpretation of the data. JUS wrote the first draft of the manuscript. All authors critically reviewed the manuscript and have approved the final version.

## Funding information

## ETHICAL APPROVAL AND CONSENT

Ethical approval was obtained from the Psychology Research Ethics committee of the University of Cambridge (Ref 2019.055).

## Supporting information

Figure S1

Table S1‐S2

Appendix S1

## Data Availability

The data that support the findings of this study are openly available in the University of Cambridge data repository (https://www.repository.cam.ac.uk/).^46^ All the data will be stored in accordance with the Data Protection Act 1998 within the University of Cambridge data for at least 10 years from the last access.
