# Supplementary figures and images for: Acceptability and potential impact on uptake of using different risk stratification approaches to determine eligibility for screening: A population‐based survey
Source: Health Expect. 2020 Dec 2;24(2):341–51. doi: 10.1111/hex.13175 (PMC8077132; doi:10.1111/hex.13175)

Appendix Figure 1

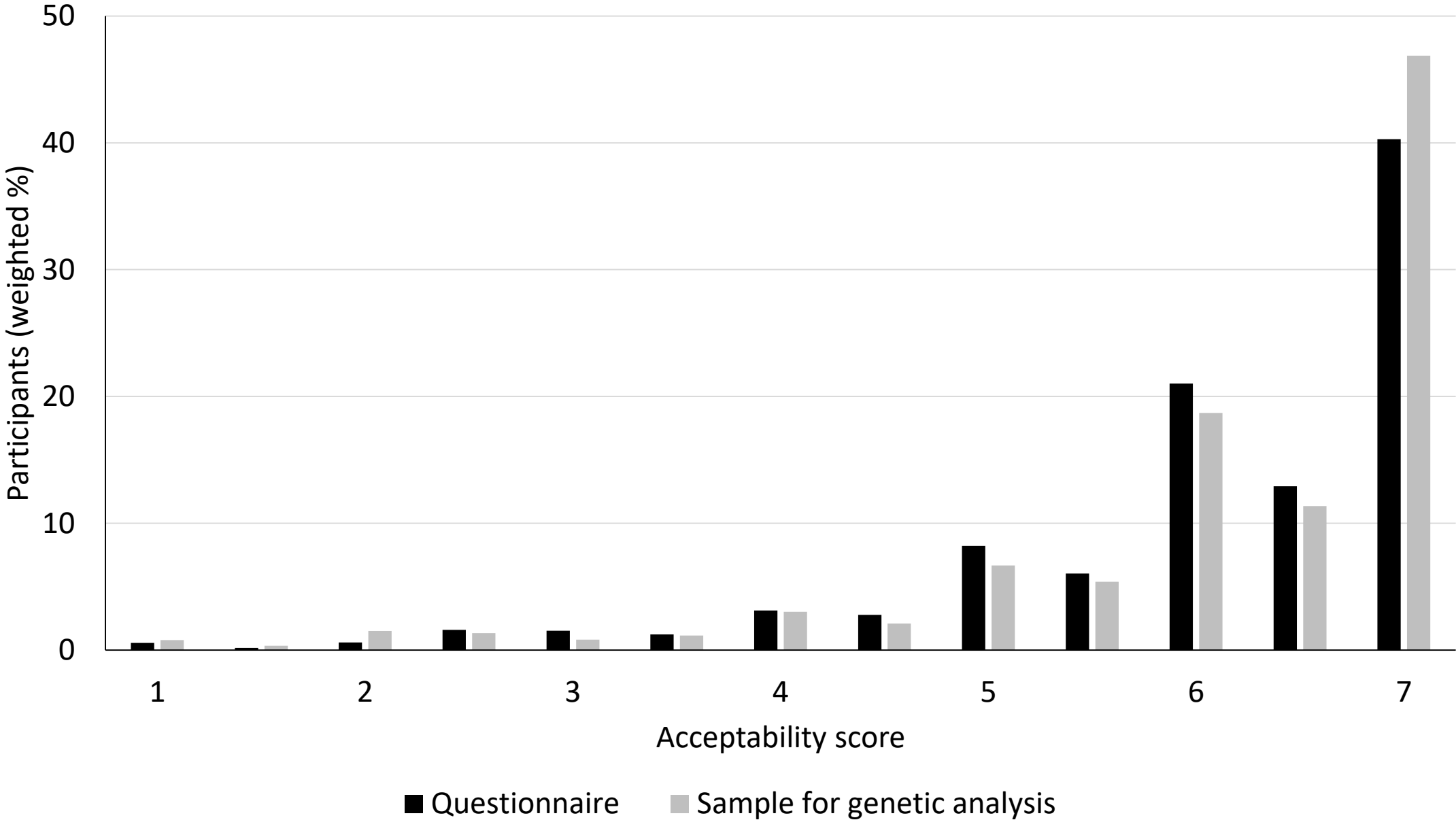

Supplement: Supplementary file 1 — Figure S1 [file HEX-24-341-s002.pdf]
