# Supplementary material for: Acceptability and potential impact on uptake of using different risk stratification approaches to determine eligibility for screening: A population‐based survey
Source: Health Expect. 2020 Dec 2;24(2):341–51. doi: 10.1111/hex.13175 (PMC8077132; doi:10.1111/hex.13175)
Supplement: Supplementary file 2 — Table S1‐S2 [file HEX-24-341-s003.docx]

**Appendix Table 1.** Multivariable analysis of comfort with waiting until older if low risk based on different criteria to determine eligibility for screening at population level. Odds ratios (OR) from multivariable ordinal logistic regression mutually adjusted for all factors in the table. Significant (p<0.05) results are shown in bold.

| **Factor** | **Sex**  Multivariable OR (95% CI) | **Age and sex†**  Multivariable OR  (95% CI) | **Simple risk score**  **(age, sex, BMI and smoking)**  Multivariable OR  (95% CI) | **Complex risk score**  **(age, sex, BMI, smoking, family history and lifestyle)**  Multivariable OR  (95% CI) | **Genetics**  Multivariable OR  (95% CI) |
| --- | --- | --- | --- | --- | --- |
| Age |  |  |  |  |  |
| 45-54 | Ref (1) | Ref (1) | Ref (1) | Ref (1) | Ref (1) |
| 55-64 | 1.04 (0.79-1.36) | 1.31 (0.88-1.96) | 1.02 (0.75-1.38) | 1.23 (0.89-1.69) | 1.03 (0.75-1.42) |
| >65 | 1.15 (0.68-1.95) | 0.90 (0.46-1.75) | **1.76 (1.07-2.91)** | **1.98 (1.25-3.15)** | **1.77 (1.11-2.82)** |
| Sex |  |  |  |  |  |
| Female | Ref (1) | Ref (1) | Ref (1) | Ref (1) | Ref (1) |
| Male | **2.05 (1.42-2.96)** | **----** | 1.24 (0.87-1.77) | 1.27 (0.89-1.80) | 1.18 (0.83-1.66) |
| Social class |  |  |  |  |  |
| ABC1 | Ref (1) | Ref (1) | Ref (1) | Ref (1) | Ref (1) |
| C2DE | 0.79 (0.48-1.29) | 0.95 (0.53-1.69) | 0.77 (0.49-1.20) | 1.02 (0.65-1.60) | 0.97 (0.64-1.47) |
| University education |  |  |  |  |  |
| No | Ref (1) | Ref (1) | Ref (1) | Ref (1) | Ref (1) |
| Yes | **1.60 (1.09-2.36)** | 1.52 (0.91-2.55) | 0.88 (0.59-1.31) | 0.95 (0.64-1.41) | 1.07 (0.73-1.56) |

**†** Female participants only

**Appendix Table 2**. Acceptability of completing a questionnaire and providing a sample for genetics in order to estimate risk. Odds ratios (OR) from ordinal logistic regression. Multivariable ordinal logistic regression mutually adjusted for all factors in the table.

| **Factor** | **Acceptability of completing a questionnaire** | | **Acceptability of providing a sample for genetics** | |
| --- | --- | --- | --- | --- |
|  | **Univariable OR**  **(95% CI)** | **Multivariable OR (95% CI)** | **Univariable OR**  **(95% CI)** | **Multivariable OR**  **(95% CI)** |
| Age |  |  |  |  |
| 45-54 | Ref (1) | Ref (1) | Ref (1) | Ref (1) |
| 55-64 | 1.13 (0.83-1.52) | 1.18 (0.87-1.61) | 0.99 (0.74-1.33) | 0.94 (0.69-1.28) |
| >65 | **2.08 (1.27-3.40)** | **2.34 (1.39-3.95)** | **1.80 (1.07-3.02)** | **2.08 (1.20-3.62)** |
| Sex |  |  |  |  |
| Female | Ref (1) | Ref (1) | Ref (1) | Ref (1) |
| Male | 1.20 (0.84-1.70) | 1.16 (0.81-1.67) | 1.04 (0.73-1.49) | 1.06 (0.73-1.51) |
| Social class |  |  |  |  |
| ABC1 | Ref (1) | Ref (1) | Ref (1) | Ref (1) |
| C2DE | 0.86 (0.55-1.35) | 1.16 (0.73-1.84) | 0.93 (0.59-1.47) | 1.01 (0.63-1.60) |
| University education |  |  |  |  |
| No | Ref (1) | Ref (1) | Ref (1) | Ref (1) |
| Yes | 1.03 (0.73-1.47) | 0.98 (0.73-1.84) | 0.96 (0.67-1.38) | 0.83 (0.60-1.22) |
