# Supplementary material for: Acceptability and potential impact on uptake of using different risk stratification approaches to determine eligibility for screening: A population‐based survey
Source: Health Expect. 2020 Dec 2;24(2):341–51. doi: 10.1111/hex.13175 (PMC8077132; doi:10.1111/hex.13175)
Supplement: Supplementary file 3 — Appendix S1 [file HEX-24-341-s001.pdf]

Acceptability and potential impact on uptake of using different risk stratification approaches to determine eligibility for screening survey

Question	Response options
[Note that this content was delivered online so the formatting looked slightly different to in this document. The participants also did not see the headings in grey boxes.]	
Thank you for agreeing to complete this questionnaire. Please answer every question. If you are uncertain about how to answer a question, then please select the closest option.	
Demographic information<br><br>*In this first section we would like to ask you a few questions about yourself. These questions allow us to make sure we are including people from a range of different backgrounds and see if different groups of people have different views. We will not be able to identify you from your answers.*	
How old are you?	• 45-49<br>• 50-54<br>• 55-59<br>• 60-64<br>• 65-69<br>• 70-74<br>• 75-79
What is your ethnic group? Choose one option that best describes your ethnic group or background.	• White<br>• Mixed/Multiple ethnic group<br>• Asian/Asian British

	• Black/African/Caribbean/Black British<br>• Other, please describe______
What is your highest education level?	• Finished school at or before the age of fifteen<br>• Completed CSEs, O-levels or equivalent<br>• Completed A Levels or equivalent<br>• Completed further education but not a degree<br>• Completed a Bachelor’s degree / Masters’ degree / PHD<br><br>Other (please specify) ______
Please indicate to which occupational group the Chief Income Earner in your household belongs, or which group fits best. This could be you: the Chief Income Earner is the person in your household with the largest income. If the Chief Income Earner is retired and has a pension please answer for their most recent occupation. If the Chief Income Earner is not in paid employment but has been out of work for less than 6 months, please answer for their most recent occupation.	• Professional or higher technical work - work that requires at least degree-level qualifications (e.g. doctor, accountant, schoolteacher, university lecturer, social worker, systems analyst)<br>• Manager or Senior Administrator (e.g. company director, finance manager, personnel manager, senior sales manager, senior local government officer)<br>• Clerical (e.g. clerk, secretary)<br>• Sales or Services (e.g. commercial traveller, shop assistant, nursery nurse, care assistant, paramedic)

	• Foreman or Supervisor of Other Workers (e.g building site foreman, supervisor of cleaning workers)<br>• Skilled Manual Work (e.g. plumber, electrician, fitter, train driver, cook, hairdresser)<br>• Semi-Skilled or Unskilled Manual Work (e.g. machine operator, assembler, postman, waitress, cleaner, labourer, driver, bar-worker, call centre worker)<br>• Other (Please specify)<br>• Have never worked
**Lifestyle and screening history**	
Lifestyle and screening history<br><br>*The following questions are about your lifestyle and your past screening decisions. Please answer as honestly as you can as this will help us with our analysis.*	
In general, would you say your health is	• Excellent<br>• Very good<br>• Good<br>• Fair<br>• Poor

What is your smoking status?	• Never smoked<br>• Used to smoke<br>• Smoke up to 20 per day<br>• Smoke 20 or more per day
What is your height?	• _____
What is your weight?	• _____
Have you ever had cancer?	• Yes<br>• No
It is important that you pay attention to this study. Please select "Strongly Disagree"	• Strongly agree<br>• Agree<br>• Neither disagree nor agree<br>• Disagree<br>• Strongly disagree
Have your parents or any brothers or sisters ever had kidney cancer?	• Yes<br>• No<br>• Don't know/prefer not to answer
**Risk stratification scenarios**	
Developing targeted screening programmes by looking at risk factors for kidney cancer	

<table>
<tr><td colspan="2">

As kidney cancer only affects about 2 in every 1000 adults, it would be very expensive to screen all adults in the UK and most of them would not benefit from the screening programme. One way of reducing the cost of screening and increasing the number of people who benefit is to only offer screening to people who we think are more likely to develop kidney cancer.

A person's risk of developing kidney cancer depends on many factors. These include:

- *Age: kidney cancer is more likely in older people. On average each year, more than a third of new cases are in people aged 75 and over.*
- *Sex: men are two to three times more likely to get kidney cancer than women*
- *Smoking: people who smoke more than 20 cigarettes per day have over double the risk of people who do not smoke at all.*
- *BMI: people who are overweight are between one third and two thirds more likely to get kidney cancer than those of a normal weight*
- *Family history: people with a parent or brother or sister with kidney cancer are two to three times more likely to get kidney cancer than people without a family history of kidney cancer*

Please consider the following scenarios and answer the questions honestly, we would like to know YOUR opinion. These all are referring to kidney cancer screening where the word screening is used.

</td></tr>
<tr><td>

Imagine how you would feel in the following scenario…

Let's say you are feeling fine. You receive a letter inviting you to kidney cancer screening as you are now aged 60. The letter states that 'experts recommend that people get their first screening test at age 60.' They then explain that 'the reason they recommend starting at 60 (rather than a younger age) is because kidney cancer does not usually occur in younger people.'

1. How reasonable does it seem to you that experts recommend using age to decide when to start screening?
2. How reasonable does it seem to you that experts recommend starting screening at the same age for all people.
3. How comfortable are you with experts using age to decide when you should start kidney cancer screening?

</td><td>

- Not at all reasonable – extremely reasonable (1-7)

</td></tr>
</table>

**SPLIT INTO MEN AND WOMEN** (participants only see one depending on their sex)<br><br>**Men**<br><br>Now imagine that you receive a letter inviting you to kidney cancer screening and it states that you are being invited because 'you are a man and men are 2-3 times more likely to develop kidney cancer than women.'<br><br>• How reasonable does it seem to you that experts would use sex to decide whether to offer kidney cancer screening?<br>• How reasonable does it seem to you that men would be offered screening and women would not?<br>• How comfortable are you with experts using sex to decide whether you should be offered kidney cancer screening?<br>• How comfortable would you be about being offered kidney cancer screening when women are not offered screening?<br><br>Now imagine that you receive a letter inviting you to kidney cancer screening as you are now aged 50. The letter states that 'experts recommend that men get their first screening test at age 50 and women at age 60.' They then explain that 'the reason they recommend starting earlier in men is because kidney cancer is 2-3 times more common in men than women'.<br><br>1. How reasonable does it seem to you that experts would use age and sex to decide what age to start kidney cancer screening?<br>2. How reasonable does it seem to you that men would be offered screening earlier than women?<br>3. How comfortable are you with experts using age and sex to decide when you should start kidney cancer screening?<br><br>**Women**	• Not at all reasonable – extremely reasonable (1-7)<br>• Not at all comfortable – extremely comfortable (1-7)

Now imagine that you did not receive an invitation but a male friend or relative who is the same age as you receives a letter inviting them to kidney cancer screening. In that letter it states that it is because 'men are 2-3 times more likely to develop kidney cancer than women.' You will not receive an invitation for screening because you are a woman.<br><br>• How reasonable does it seem to you that experts would use sex to decide whether to offer kidney cancer screening?<br>• How reasonable does it seem to you that men would be offered screening and women would not?<br>• How comfortable are you with experts using sex to decide whether you should be offered kidney cancer screening?<br>• How comfortable would you be about not being offered kidney cancer screening when men are offered screening?<br><br>Now imagine that when you are 60 you also receive a letter. In that it states that 'experts recommend that men get their first screening test at age 50 and women at age 60.' They then explain that 'the reason they recommend starting earlier in men is because kidney cancer is 2-3 times more common in men than women'.<br><br>• How reasonable does it seem to you that experts would use age and sex to decide what age to start kidney cancer screening?<br>• How reasonable does it seem to you that men would be offered screening earlier than women?<br>• How comfortable are you with experts using age and sex to decide when you should start kidney cancer screening?<br>• How comfortable would you be about having to wait until you were older than your male friend or relative to start screening?	
Now imagine that you receive a different letter inviting you to screening. In that it describes how experts have developed a calculator to estimate how likely an individual is to develop kidney cancer. This calculator, which is based on scientific research studies, uses information available from routine GP	• Not at all reasonable – extremely reasonable (1-7)

records, such as your age, sex, BMI and smoking status, to identify at what age you should first have kidney cancer screening. Like the screening tests, it is not 100% reliable. Someone who is estimated to be at higher risk based on the calculator is not destined to get kidney cancer. A low risk estimate also does not mean someone is completely without risk of getting kidney cancer.<br><br>• How reasonable does it seem to you that experts would use a calculator that includes these factors to predict each person's risk of kidney cancer and use that to decide what age to start kidney cancer screening?<br>• How reasonable does it seem to you that people at higher risk based on this calculator would be invited for kidney cancer at a younger age than people at lower risk?<br>• How comfortable are you with experts using a kidney cancer risk calculator based on these factors to decide what age you should start kidney cancer screening?<br>• How comfortable would you be about having to wait until you were older to start screening if you were low risk based on this calculator?<br><br>Imagine that the letter says that you are being invited now because you are at higher risk than the average person in England.<br><br>• How would being told you are high-risk influence your decision to attend screening?<br><br>Now imagine you are older and you receive a letter that says you are being invited and you are at lower risk than the average person in England.<br><br>• How would being told you are low-risk influence your decision to attend screening?	• Not at all comfortable – extremely comfortable (1-7)<br><br>• Much less likely to attend (1)<br>• Slightly less likely to attend<br>• No influence<br>• Slightly more likely to attend<br>• Much more likely to attend (5)
Now imagine that along with the letter you are asked to fill out a questionnaire which asks about your family history of cancer and your lifestyle, including how much physical activity you do and your diet. This information can then be used along with your age, sex, BMI and smoking status in a new calculator to estimate your risk of developing kidney cancer in order to determine at what age you should be offered screening. The relationship of these factors with the risk of kidney cancer is based on scientific research. Like the calculator in the previous section, this new calculator is not 100% reliable. Someone	• Not at all reasonable – extremely reasonable (1-7)<br>• Not at all comfortable – extremely comfortable (1-7)

who is estimated to be at higher risk based on the calculator is not destined to get kidney cancer. A low risk estimate also does not mean someone is completely without risk of getting kidney cancer.<br><br>• How reasonable does it seem to you that doctors would require you to fill out a questionnaire to predict your risk of kidney cancer?<br>• How comfortable would you be filling in the questionnaire and giving information about your family history and lifestyle?<br>• How reasonable does it seem to you that experts would use a calculator that includes these factors to predict each person's risk of kidney cancer and use that to decide what age to start kidney cancer screening?<br>• How reasonable does it seem to you that people at higher risk based on this calculator would be invited for kidney cancer at a younger age than people at lower risk?<br>• How comfortable are you with experts using a kidney cancer risk calculator based on these factors to decide what age you should start kidney cancer screening?<br>• How comfortable would you be about having to wait until you were older to start screening if you were low risk based on this calculator?	
Having certain genes can also increase your risk of kidney cancer. Imagine that you are invited to be tested for these high-risk genes by providing a sample of blood or a cheek swab. This information would then be used to calculate how likely you are to develop kidney cancer and used to decide what age to start screening. The relationship of these genes with the risk of kidney cancer is based on scientific research. Like the risk calculators above, the calculator based on genes is not 100% reliable. Someone who is estimated to be at higher risk based on their genes is not destined to get kidney cancer. A low risk estimate also does not mean someone is completely without risk of getting kidney cancer.<br><br>• How reasonable does it seem to you that doctors would require you to provide a sample of blood or a cheek swab to predict your risk of kidney cancer?<br>• How comfortable would you be providing a sample of blood or a cheek swab to allow experts to test whether you have high-risk genes?	• Not at all reasonable – extremely reasonable (1-7)<br>• Not at all comfortable – extremely comfortable (1-7)<br><br>• Much less likely to attend (1)<br>• Slightly less likely to attend<br>• No influence<br>• Slightly more likely to attend<br>• Much more likely to attend (5)

• How reasonable does it seem to you that experts would use a genetic test to predict each person's risk of kidney cancer and use that to decide what age to start kidney cancer screening?<br>• How reasonable does it seem to you that people at higher risk based on their genes would be invited for kidney cancer at a younger age than people at lower risk?<br>• How comfortable are you with doctors using a genetic risk calculator to decide what age you should start kidney cancer screening?<br>• How comfortable would you be about having to wait until you were older to start screening if you were low risk based on a genetic risk calculator?<br><br>Imagine that the letter says that you are being invited now because based on your genes you are at higher risk than the average person in England.<br><br>• How would being told you are high-risk based on your genes influence your decision to attend screening?<br>• If instead, the letter said you were being invited now because you are at lower risk than the average person in England, how would being told you are low-risk based on your genes influence your decision to attend screening?	
Thank you for completing the survey. Your time and responses are very much appreciated.	
